# Retinal layers and visual conductivity changes in a case series of microangiopathic ischemic stroke patients

**DOI:** 10.1186/s12883-020-01894-y

**Published:** 2020-09-03

**Authors:** John-Ih Lee, Lena Gemerzki, Margit Weise, Laura Boerker, Jonas Graf, Lea Jansen, Rainer Guthoff, Orhan Aktas, Michael Gliem, Sebastian Jander, Hans-Peter Hartung, Philipp Albrecht

**Affiliations:** 1 Department of Neurology, Medical Faculty, Heinrich-Heine-University, Duesseldorf, Germany; 2 Department of Ophthalmology, Medical Faculty, Heinrich-Heine-University, Duesseldorf, Germany

**Keywords:** Optical coherence tomography, Multifocal visual evoked potentials, Ischemic stroke, Microangiopathy

## Abstract

**Background:**

It is unknown whether microangiopathic ischemic strokes outside the visual pathway go along with subclinical changes of the retinal structure or the visual system. The objectives of this prospective non-interventional case series were to investigate if spectral-domain optical coherence tomography (SD-OCT) or multifocal visual evoked potentials (mfVEPs) can detect structural retinal changes or functional impairment of the visual system in patients with microangiopathic ischemic stroke.

**Methods:**

We used SD-OCT to cross-sectionally analyze the retinal morphology of 15 patients with microangiopathic ischemic stroke according to the Trial of Org 10172 in Acute Stroke Treatment (TOAST) classification not affecting the visual pathway. We employed semi-automated segmentation of macular volume scans to analyze the thickness of the macular retinal layers and peripapillary ring scans to investigate the retinal morphology in comparison to a control group without stroke. Visual function was assessed by the mfVEP technique in 13 microangiopathic ischemic stroke patients.

**Results:**

First peak latency of mfVEPs was significantly delayed in the microangiopathic ischemic stroke group compared to the control patients. Neither the retinal layers nor the mfVEPs’ amplitude differed between the microangiopathic ischemic stroke patients and the control group.

**Conclusions:**

In conclusion, microangiopathic ischemic stroke patients presented a delayed first peak latency in mfVEPs as a sign of subclinical functional impairment of the visual pathway. However, our case series suggests no influence on retinal structure resulting from microangiopathic ischemic stroke outside the visual system. Larger and longitudinal studies are needed to confirm these mfVEP findings.

## Background

Ischemic stroke is among the most common reasons for years of life lost (YLL) [1]. Microangiopathy in the form of small vessel occlusion is a common etiology of ischemic stroke. Arterial hypertension and diabetes mellitus belong to the most important vascular risk factors for cerebral microangiopathic infarctions [2, 3]. Furthermore, arterial hypertension and diabetes mellitus are major risk factors for retinopathy, as they may also affect the small vessels of the retina [4, 5]. Since there are effective treatment options, detection and therapeutic modification of these vascular risk factors are essential for stroke prevention [6, 7]. We hypothesized that patients with microangiopathic cerebral infarctions may also have subclinical structural and/or functional abnormalities of the retina or the visual pathway below the threshold of permanent clinical symptoms. Structural retinal layer abnormalities can be non-invasively assessed by spectral domain optical coherence tomography (SD-OCT) [8–11]. SD-OCT allows a reliable measurement of the different retinal layers and can detect retinal axonal and neuronal loss even in the absence of overt visual symptoms in other diseases [8–14]. Visual evoked potentials (VEPs) are suited to investigate functional impairment of the entire visual pathway [15–17], and multifocal VEPs (mfVEPs) have demonstrated a higher sensitivity for pathology compared to fullfield VEPs, in previous studies [15–17]. The advantage of the mfVEP technique is that by stimulating the different sectors of the visual field consecutively (instead of all at once as during full field VEP (ffVEP)) the cancellation artefacts of electric dipoles reaching the cortex at the same time are avoided. Furthermore, the mfVEP covers a wider angle of the visual field (eccentricity up to 24**°**). Therefore, mfVEP has a much higher sensitivity for changes as compared to ffVEP, which has also been demonstrated in clinical studies [18]. The aim of our case series was to cross-sectionally analyze subclinical differences in retinal layers and/or visual function in eyes of microangiopathic ischemic stroke patients according to the Trial of Org 10172 in Acute Stroke Treatment (TOAST) classfication [19] without permanent clinical deficit in the visual pathway.

## Methods

### Ethics

The local ethics committee of Heinrich Heine University Duesseldorf approved this observational case series (registration number 4436R). Written informed consent was obtained from all participants in accordance with the Declaration of Helsinki.

### Patients

Patients were consecutively recruited from the stroke unit at the Department of Neurology, Heinrich- Heine-University Duesseldorf, Germany, between 2013 and 2016. Inclusion criteria were ischemic stroke with microangiopathic etiology according to the TOAST classification [19] without clinically affecting the visual pathway as assessed by patient interview, confrontational visual field testing and mfVEPs. Other stroke etiologies and ischemic stroke affecting the visual pathway diagnosed by computed tomography (CT) or magnetic resonance imaging (MRI) were excluded. All patients underwent a neuro-ophthalmologic examination including slit lamp examination, tonometry and ophthalmoscopic fundus imaging. Exclusion criteria were relevant ophthalmologic and systemic diseases with potential influence on retinal morphology as defined by the OSCAR-IB criteria [20] and any cerebral lesions within the visual pathway. Corrected visual acuity was assessed using early treatment of diabetic retinopathy study (ETDRS) charts. Patients with disorders of the peripheral nervous system like mononeuropathy, non- diabetic polyneuropathy, lumbosacral plexopathy, and somatoform disorders were selected for the control group. Patients with neurodegenerative, inflammatory diseases of the central nervous systems, stroke, cerebral lesions within the visual pathway, relevant ophthalmologic and systemic diseases with potential influence on retinal morphology as defined by the OSCAR-IB criteria [20] were not eligible for the control group.

**Fig. 1:**
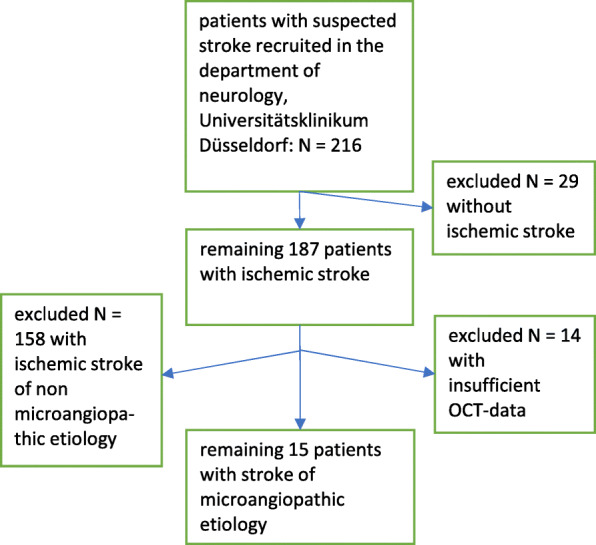
Flow chart of the inclusion/exclusion process. Out of 216 patients with suspected stroke, 201 patients were excluded (29 patients without ischemic stroke, 158 patients with non microangiopathic ischemic stroke etiologies and 14 patients with insufficient SD-OCT data). 15 patients microangiopathic ischemic stroke patients were eligible for analysis

Out of 216 patients recruited from our stroke unit 15 patients with microangiopthic ischemic stroke remained in our analysis. For SD-OCT analysis they were compared to control patients. For details see the recruitment flow chart (Fig. 1).

### Ultrasound

Intima media thickness (IMT) was measured at the far wall side of both common carotid arteries (CCAs) 2 cm below the distal end of CCA [21, 22] by ultrasound using a Toshiba ultrasound system (Aplio XG, Xario) investigated with the linear probe (7,5 MHz).

### SD-OCT methodology

SD-OCT methodology and results are reported in line with the APOSTEL reporting recommendations [23], methods have also been used and described elsewhere [8–10, 24]. In short, 61 vertical scans centered on the fovea (30° × 25°, high speed scanning mode) were performed for macular volume scans. In addition, 12° peripapillary ring scans centered on the disc (high-resolution scanning mode) were obtained. For SD-OCT imaging SPECTRALIS OCT device (Heidelberg Engineering, Germany) with the help of the image alignment eye-tracking software system (TruTrack and Nsite analytics, Heidelberg Engineering) of both eyes was used. Averaging of macular volume scans was performed from 14 images and of peripapillary ring scans from 100 scans (Automatic Real Time, ART). The image quality threshold was above 20 dB. Retinal layers were segmented semi-automatically with manual correction of errors using the Heidelberg Eye Explorer software (version HEYEX 1.8.6.0, Viewing Module 5.8.3.0). Ganglion cell layer (GCL) and the inner plexiform layer (IPL) were measured together as the ganglion cell/inner plexiform layer complex (GCIP). A blinded rater (LG) checked all scans for correct segmentation and manually adjusted segmentation errors. Only scans meeting the OSCAR-IB quality control criteria [20] were used for analysis. As a result, not all scans of all patients were suitable for analysis. Layer volumes of the retinal layers and the total retinal volume (TRV) were measured using the mean volume of all sectors of the standard 1, 3, 6 mm ETDRS grid in macular volume scans.

### mfVEP methodology

mfVEPs assessing the visual pathway function were recorded by the Visionsearch mfVEP device according to the manufacturer’s instructions as previously used and described [24–26]. In brief, for monocular stimulation the Visionsearch device applied simultaneous multi-focal stimulation of a cortically scaled dartboard pattern of 56 segments (Michelson contrast, 99%) of the visual field (24 degrees of eccentricity) with a 68 s pseudorandom sequence. A 2-channel visual response was recorded with the support of a custom designed occipital cross electrode holder, determining the position of the four occipital electrodes 4.5 centimeters (cm) lateral on each side and 4 cm above as well as 5.5 cm below the inion. A forehead holder was used to assure a viewing distance of 30 cm. Participants were refracted for near vision. Fixation was controlled using an active focusing target displaying arrows pointing in varying directions and asking patients to indicate the direction using a trigger. Runs with < 90% correct trigger answers were repeated. We used a maximum of 12 single 68-s monocular measurements for each eye. For each channel, the largest peak-to-trough amplitude within the interval of 70 to 210 ms was selected. The first peak of the largest wave for each segment was automatically determined for latency measurement. For the final analysis mean values of amplitude and latency were calculated by averaging the amplitude and latency of the individual sectors as previously described with the support of the TERRA Software 1.6.21 [25, 27].

### Statistical evaluation

Statistical analyses were performed using SPSS Statistics 20 (IBM). For analysis of the demographics dichotomized parameters were compared by use of Fisher’s exact test while continuous data were analyzed with Mann-Whitney-U test. For eye data, generalized estimation equation models (GEE) accounting for within subject inter-eye correlations using an exchangeable working correlation matrix and correcting for age and gender were applied to analyze associations between SD-OCT parameters and mfVEP parameters and to test for differences between microangiopathic ischemic stroke patients and controls. As several retinal layers, mfVEP amplitudes and latencies were analyzed in this exploratory analysis, Bonferroni correction for multiple testing was performed. The association between IMT and retinal layers was investigated by Spearman correlation analysis.

Subjects with missing data were excluded from the respective analysis, *p*-values < 0.05 were considered significant.

## Results

### Patients

15 patients and 22 control patients were included according to our inclusion and exclusion criteria. Examinations were obtained with a mean of 5 days (+/− 2.6 standard deviation (SD)) after the ischemic stroke event. A subgroup of 13 microangiopathic ischemic stroke patients and 13 control patients were examined by mfVEP. Baseline parameters and vascular risk factors are presented in Table 1. The age differed significantly between both groups (Mann Whitney U test *p* < 0.05) with older patients in the microangiopathic ischemic stroke group. The vascular risk factors except for diabetes mellitus, coronary heart disease were also significantly more frequent (Fisher’s exact test with *p* < 0.05) in the microangiopathic ischemic stroke group. Mean corrected visual acuity was significantly reduced in the microangiopathic ischemic stroke group compared to the control group. The intraocular pressure was normal (< 20 mmHg) in all patients and slit lamp examination revealed no pathologies. In the mfVEP subgroup, the 13 control patients had significantly less smokers and less hyperlipidemia compared to the 13 microangiopathic ischemic stroke patients (Fisher’s exact test *p* < 0.05). Available arterial blood pressure values of our microangiopathic ischemic stroke patients (*n* = 15) with a median of 130 mmHg (IQR 120–145) systolic and a median of 70 mmHg diastolic (IQR 60–80) values compared to the control patients (*n* = 13) with a median of 120 mmHg (IQR 105–135) systolic and a median of 65 mmHg (IQR 60–72.5) diastolic values at the day of SD-OCT and mfVEP examination revealed no significant difference in systolic or diastolic values (Mann Whitney U Test, n.s.).

**Table 1:** Baseline parameters of 15 patients with microangiopathic ischemic stroke and 22 control group persons and the mfVEP subgroup of 13 microangiopathic ischemic stroke patients and 13 controls. Absolute numbers and percentages or median with interquartile range are provided for demographics, risk factors and visual acuity

	SD-OCT			mfVEP		
	*N* = 15 (microangiopathic ischemic stroke)	*N* = 22 (control)	Test	*N* = 13 (microangiopathic ischemic stroke)	*N* = 13 (control)	Test
Median age in years (interquartile range), [age range]	63 (50–71), [45–79]	53 (46–55), [21–80]	Mann Whitney U test *p* < 0.05	63 (53.5–73), [45–79]	55 (48.5–66.5), [44–80]	Mann Whitney U test n.s.
Male gender	10 (67%)	13 (59%)	Fisher’s exact test n.s.	9 (69%)	10 (77%)	Fisher’s exact test n.s.
Arterial Hypertension	12 (80%)	4 (18%)	Fisher’s exact test *p* < 0.05	10 (77%)	2 (15%)	Fisher’s exact test n.s.
Diabetes mellitus	5 (33%)	2 (9%)	Fisher’s exact test n.s.	3 (23%)	2 (15%)	Fisher’s exact test n.s.
Vascular disease	4 (27%)	0 (0%)	Fisher’s exact test *p* < 0.05	4 (31%)	0 (0%)	Fisher’s exact test n.s.
Smoking	10 (67%)	1 (5%)	Fisher’s exact test *p* < 0.01	9 (69%)	1 (8%)	Fisher’s exact test *p* < 0.01
Coronary heart disease	2 (13%)	0 (0%)	Fisher’s exact test n.s.	2 (15%)	0 (0%)	Fisher’s exact test n.s.
Hyperlipidemia	10 (67%)	3 (14%)	Fisher’s exact test *p* < 0.01	9 (69%)	3 (23%)	Fisher’s exact test *p* < 0.01
Median corrected visual acuity in log MAR units (interquartile range)	0.01 (−0.05 to 0.07) (*N* = 28 eyes)	0.00 (− 0.12 to + 0.12) (*N* = 16 eyes)	Mann Whitney U test *p* < 0.05	0.01 (0 to + 0.1) (*N* = 24 eyes)	0.00 (− 0.1 to + 0.1) (*N* = 14 eyes)	Mann Whitney U test n.s.

### Secondary prevention

All ischemic stroke patients were treated with best medical treatment according to the established recommendations for secondary stroke prevention of the German Neurological Society [28].

### SD-OCT findings

Complete SD-OCT scans of both eyes were obtained from all patients and revealed no structural abnormalities in any of the retinal layers or in the pigment epithelium in any of the subjects meeting the inclusion criteria (data provided in Table 2).

**Table 2:** SD-OCT findings. SD-OCT analysis considering available 14 patients (*n* = 28 eyes) and 19 controls (*n* = 38 eyes) with macular scans as well as 15 patients (*n* = 30 eyes) and 18 controls (*n* = 36 eyes) with peripapillary scans: Means, standard deviations and *p*-values are provided for each macular retinal layer volume and the peripapillary RNFL (pRNFL) thickness at baseline, comparing microangiopathic ischemic stroke patients to the control group, n.s. indicating no significant difference. *P*-values < 0.05 were considered as statistically significant (GEE analysis with Bonferroni correction for multiple testing)

	Mean value of microangiopathic ischemic stroke patients	+/−SD	Mean value of the control group	+/−SD	***p***-value
**RNFL (mm**^**3**^**)**	0.9293	0.17378	0.9061	0.0950	n.s.
**GCIP (mm**^**3**^**)**	0.9721	0.11236	1.0424	0.0618	n.s.
**IPL (mm**^**3**^**)**	0.8029	0.08146	0.8568	0.0505	n.s.
**INL (mm**^**3**^**)**	0.9579	0.09061	0.9211	0.0417	n.s.
**OPL (mm**^**3**^**)**	0.7750	0.03677	0.8016	0.0593	n.s.
**ONL (mm**^**3**^**)**	1.7968	0.14810	1.7874	0.1466	n.s.
**RPE (mm**^**3**^**)**	0.3986	0.04369	0.4053	0.0278	n.s.
**PR (mm**^**3**^**)**	2.2432	0.07339	2.2363	0.0613	n.s.
**TRV (mm**^**3**^**)**	8.4768	0.53613	8.5534	0.2172	n.s.
**pRNFL (μm)**	56.2000	22.76628	56.5556	11.0981	n.s.

**Fig. 2:**
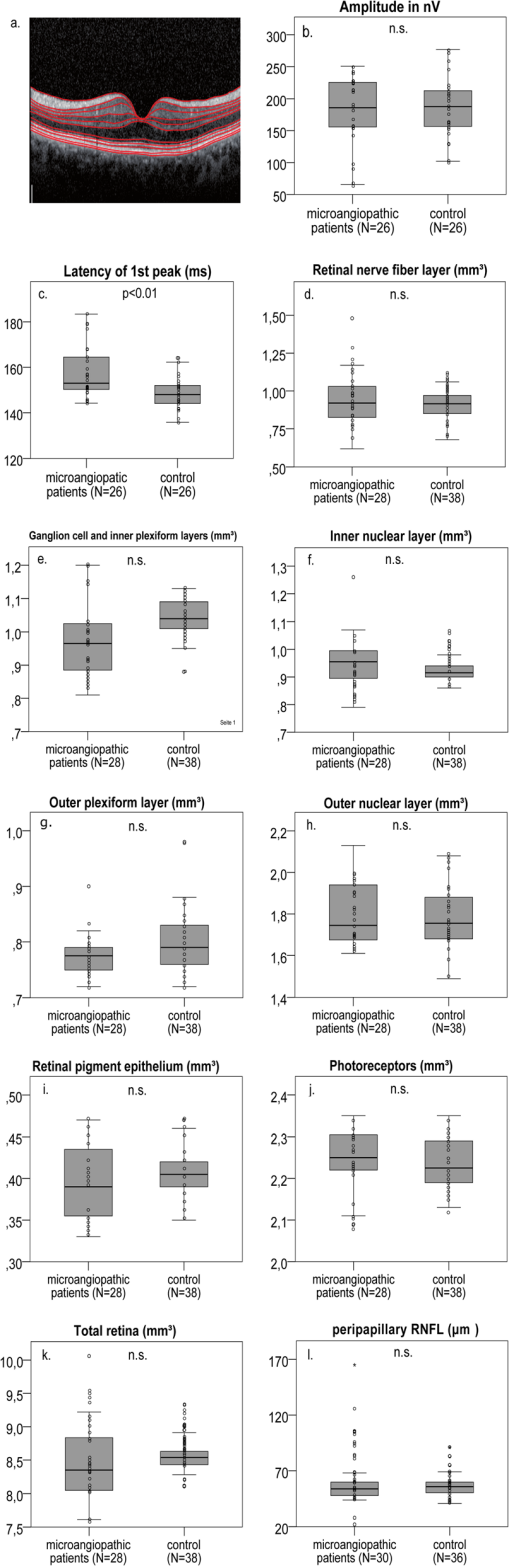
Example macular SD-OCT layer scan, SD-OCT and mfVEP parameters in microangiopathic ischemic stroke patients. An example macular volume SD-OCT layer scan is demonstrated through the fovea centralis with the red lines indicating the boundaries of the different retinal layers (**a**). In mfVEPs, amplitude (**b**) and first peak latency (**c**) between the microangiopathic ischemic stroke patients and the control group were analyzed. SD-OCT layers were measured in volume scans centered on the fovea and pRNFL was measured in peripapillary ring scans centered on the optic disc (**d**-**l**). The analysis revealed no significant difference of the retinal layer volume or thickness between the microangiopathic ischemic stroke patients and the control group. **b**.-**l** Boxplots with scatter plots are demonstrated for the microangiopathic ischemic stroke patients and the control group with n.s. indicating no significant difference. *P*-values < 0.05 were considered as statistically significant (GEE analysis with Bonferroni correction for multiple testing). The horizontal line in the boxplots represents the medians, the box the interquartile range (IQR) and the whiskers the minimum and maximum values (excluding outliers). Outliers (1.5–3.0 times outside the IQR) are presented as circles and extreme outliers (> 3.0 times outside the IQR) are presented as asterisks

We observed no significant differences of the measured retinal layers (Fig. 2 d.-l. and Table 2) between the total cohort of 15 microangiopathic ischemic stroke patients and the control group of 19 persons.

### MfVEP findings

We obtained mfVEPs from 13 microangiopathic ischemic stroke patients (*n* = 26 eyes) and 13 control persons (*n* = 26 eyes). A significant delay of the first peak latency was detected in microangiopathic ischemic stroke patients, while amplitudes did not differ (Fig. 2 b.-c. and Table 3).

**Table 3:** MfVEP findings. Means and standard deviations as well as *p*-values for the comparison of mfVEP first peak latencies in ms and amplitudes in nV are provided comparing microangiopathic ischemic stroke patients with the control group. Analyses were performed for all available 13 patients (*n* = 26 eyes, age range from 45 to 79 years) and 13 control persons (*n* = 26 eyes, age range from 44 to 80 years). N.s. indicates no significant difference and *p*-values < 0.05 were considered as statistically significant (GEE analysis with Bonferroni correction for multiple testing)

	Microangiopathic ischemic stroke patients			Control group			***p***-value
	Mean value +/−SD	Median value	Range	Mean value +/−SD	Median value	Range	
**Amp (nV)**	178.1773 +/− 56.0464	186.0200	65.71–250.47	187.7050 +/− 46.7700	187.7050	102.00–276.79	n.s.
**1st peak latency (ms)**	158.9197 +/− 10.6981	152.9800	144.19–183.42	148.0000 +/− 6.8500	148.0000	135.78–164.11	p < 0.01

Both eyes of the microangiopathic stroke patients showed an increased mean first peak mfVEP latency of 157.96 ± 11.87 SD ms for the left eyes and a mean of 157.47 ± 11.66 SD ms for the right eyes compared to the control group with a mean of 148.61 ± 6.79 SD ms for the left eyes and a mean of 148.58 ± 7.19 SD ms for the right eyes (Mann Whitney U test, *p* < 0.05). No significant difference of first peak latency between the left and right eyes of the microangiopathic ischemic stroke patients or the control patients was detected (Wilcoxon test, n.s.)

**Fig. 3:**
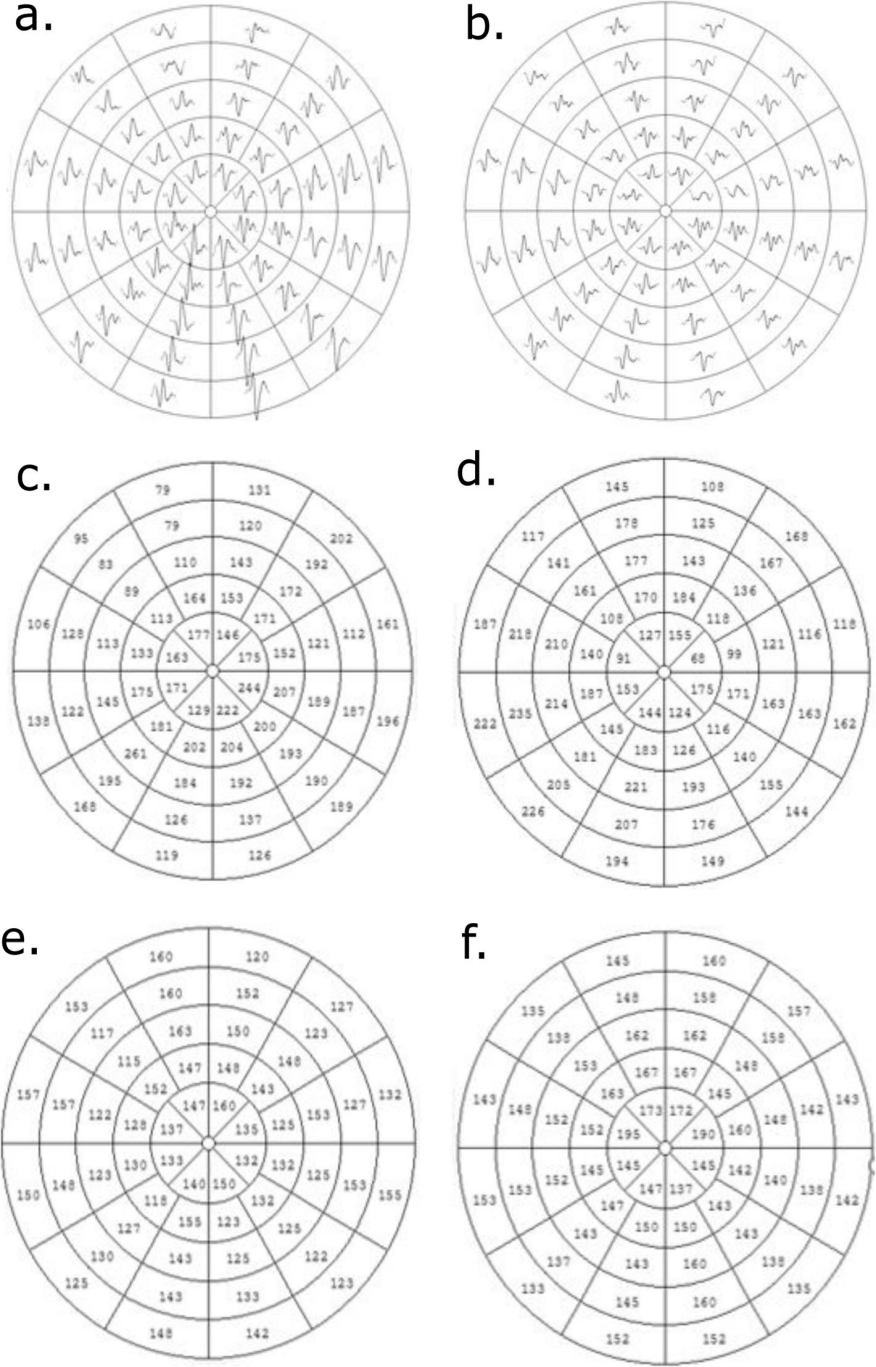
Example left eye mfVEPs of a control person (**a**., **c**. and **e**) and a microangiopathic ischemic stroke patient (**b**., **d**. and **f**.): a. mfVEP responses of a control person. **b**. mfVEP responses of a microangiopathic ischemic stroke patient. **c**. mfVEP amplitudes of a control person. **d**. mfVEP amplitudes of a microangiopathic ischemic stroke patient. **e**. mfVEP first peak latencies of a control person. **f**. mfVEP first peak latencies of a microangiopathic ischemic stroke patient

None of the 26 eyes of the microangiopathic ischemic stroke patients and controls showed a specific pattern of mfVEP abnormality defined as ≥ three adjacent sectors with more than two standard deviations of first peak latency delay or amplitude reduction. An example of mfVEP responses, amplitudes and first peak latencies of a control patient and a microangiopathic ischemic stroke patient are presented in Fig. 3.

The microangiopathic ischemic stroke group was significantly older than the control group. However, we statistically corrected for age and the GEE analysis revealed no significant (*p* > 0.05) effect of age on mfVEP first peak latencies. Two patients in the microangiopathic stroke group had delayed mfVEP first peak latencies defined as > 2 standard deviations above controls. We observed no specific pattern regarding the location of delays.

### IMT findings

In the 9 microangiopathic stroke patients (mean IMT of the right and left side, *N* = 18 eyes with 0.7167 mm +/− 0.15435 mm SD) available for analysis, no significant correlation (Spearman correlation) between IMT of the right or left CCA and retinal layer volume or thickness in SD-OCT or mfVEP parameters (first latency peak and amplitude) was identified (data not shown).

## Discussion

In this case series, we investigated if microangiopathic ischemic stroke according to the TOAST classification [19] is associated with changes in retinal morphology detectable by SD-OCT using a cross-sectional prospective design. A strength of our case series was that not only structure (SD-OCT) but also function (mfVEPs) were investigated. In summary, in mfVEPs a significant delay of the first peak latency of microangiopathic ischemic stroke patients compared to the control group was observed. However, our results demonstrate that there were no relevant differences in retinal layer volume or thickness between microangiopathic cerebral ischemia patients and controls.

Previous SD-OCT studies have reported a reduction of the RNFL in cerebrovascular disorders like asymptomatic internal carotid artery stenosis [29] and moyamoya angiopathy [11]. But so far, to our knowledge there is only very little data on SD-OCT and mfVEP changes in microangiopathic ischemic stroke patients. Xu et al. [30] found an association between arterial hypertension, which is an important risk factor for microangiopathic ischemic stroke, and localized retinal nerve fiber layer defects (RNFLDs) on fundus photographs, while Wang et al. [31] discovered a strong association of RNFLDs detected by SD-OCT in patients with previous or acute ischemic stroke and vice and versa. But in their observation Wang et al. [31] found no significantly associated prevalence of localized RNFLDs with the different stroke subgroups according to the TOAST classification [19]. Regarding cerebral microangiopathy Kim et al. [32] detected a higher prevalence of RNFLDs in fundus photographs of hypertensive, cerebral small vessel disease (SVD) based on MRI, male and older patients. Interestingly, among the cases of cerebral SVD lacunar infarctions, as in our case series, were not significantly associated with RNFLDs, while white matter lesions (WMLs) were [32].

In our mfVEP analysis averaging the amplitudes and latencies of the 56 mfVEP responses for each of the 13 microangiopathic ischemic stroke patients had significant delayed first peak latencies compared to the control group. This finding should be interpreted with caution as the microangiopathic ischemic stroke group was significantly older than the control group and recent as well as earlier studies reported an increase of VEP latencies with age [33–35]. However, in our analysis, age was not a significant predictor for a first peak latency in mfVEPs in the microangiopathic ischemic stroke group. Therefore, a more probable explanation may be general cerebral microangiopathic ischemic changes leading to impairment of the cerebral visual pathway detected by the highly sensitive mfVEP methodology [18]. This is in line with a previous study by Hacke et al. [36], who described delayed latencies of VEPs in 36% of patients with subcortical arteriosclerotic encephalopathy and on a group level significant differences compared to healthy controls. A limitation of our mfVEP study is that mfVEP repeatability was not assessed. However, previous investigations have reported a good reproducibility of first peak latency in mfVEP [37].

The negative findings of our SD-OCT analyses, which only partly contradicts a previous report by Wang and colleagues [31], might be explained by the small sample size, different ethnicity of the group (Caucasian versus Asian) and the analyses of only microangiopathic ischemic strokes and not ischemic strokes of all etiologies compared to the study of Wang et al. [31]. This is supported by the observation that Wang et al. also did not find that the prevalence of localized RNFLDs was significantly associated with the different TOAST subgroups. Furthermore, they did not specifically address SD-OCT findings of microangiopathic ischemic stroke patients. However, our findings are in agreement with the observations of Kim et al. [32], who could not find any significant change in RFNLDs or retinal layers in patients with lacunar infarctions as well. In order to detect small changes, bigger cohort sizes and longitudinal examinations may be required.

## Conclusion

Our case series provides no evidence for influence of microangiopathic ischemic stroke outside the visual system on retinal layer thickness assessed by SD-OCT. mfVEP, on the other hand, can give evidence of subclinical visual pathway damage in patients with microangiopathic stroke. To exclude subtle changes in SD-OCT and to confirm the observed mfVEP changes further larger studies with longitudinal design including a long follow up period with a healthy age and gender matched control group would be needed. However, the magnitude of such subtle changes in SD-OCT is likely to be below the accuracy of the measurements and therefore very unlikely to be meaningful in a clinical setting.

## Data Availability

The datasets used and/or analysed during the current case series are available from the corresponding author on reasonable request.

## Abbreviations

CCA: Common carotid artery
CT: Computed tomography
ETDRS: Early treatment of diabetic retinopathy study
GCIP: Ganglion cell/inner plexiform layer complex
GCL: Ganglion cell layer
GEE: Generalized estimation equation models
IMT: Intima media thickness
INL: Inner nuclear layer
IPL: Inner plexiform layer
MRI: Magnetic resonance imaging
mfVEPs: Multifocal visual evoked potentials
n.s.: Non-significant
OCT: Optical coherence tomography
ONL: Outer nuclear layer
OPL: Outer plexiform layer
PR: Photoreceptors
pRNFL: Peripapillary RNFL
RNFL: Retinal nerve fiber layer
RNFLDs: Retinal nerve fiber layer defects
RPE: Retinal pigment epithelium
SD: Standard deviation
SD-OCT: Spectral-domain optical coherence tomography
SVD: Small vessel disease
TOAST: Trial of Org 10172 in Acute Stroke Treatment
TRV: Total retinal volume
VEPs: Visual evoked potentials
